# Longitudinal association between insulin resistance and depressive symptoms in older adults: the mediating role of inflammation in the ELSA cohort

**DOI:** 10.3389/fpsyt.2026.1848018

**Published:** 2026-08-12

**Authors:** Yiming Ma, Zhiyong Hou, Bei Yao, Ran Jia, Yanan Yu, Jun Liu, Zhong Wang

**Affiliations:** Institute of Basic Research in Clinical Medicine, China Academy of Chinese Medical Sciences, Beijing, China

**Keywords:** C-reactive protein (CRP), depressive symptoms, estimated glucose disposal rate (eGDR), longitudinal study, mediation analysis

## Abstract

**Background:**

Insulin resistance (IR) has been implicated in depression, yet the dynamic interplay between concurrent changes in IR and depressive symptoms over time, and whether systemic inflammation mediates this relationship, remains unclear.

**Methods:**

Data were obtained from 3,404 older adults (aged ≥50 years) in the English Longitudinal Study of Ageing (ELSA) across Waves 2, 4, and 6. IR was assessed using the estimated glucose disposal rate (eGDR). Depressive symptoms were measured with the CES-D scale (0-8). C-reactive protein (CRP) was log-transformed as the mediator. Linear mixed models were used to assess the longitudinal association between changes in eGDR and CES-D score. Restricted cubic splines were applied to explore potential nonlinear dose-response relationships. Mediation analysis was conducted to test whether CRP mediated the association. Subgroup and sensitivity analyses were performed to evaluate the robustness of the findings.

**Results:**

In the overall population, eGDR was significantly associated with CES-D score (P = 0.003), with no evidence of nonlinearity (P = 0.074). The overall eGDR × time interaction was not significant (P = 0.349). When stratified by joint trajectories of eGDR and CES-D, significant eGDR × time interactions were observed in two groups: the decrease-increase group (eGDR declined and CES-D increased, β = –0.018, P < 0.001), and the increase-decrease group, (eGDR increased and CES-D decreased, β = 0.021, P < 0.001). CRP partially mediated the association, with an indirect effect of -0.00092 (95% CI: -0.00155 to -0.00042), accounting for 10.8% of the total effect. Subgroup and sensitivity analyses confirmed robustness.

**Conclusions:**

The longitudinal association between IR and depressive symptoms differs across developmental trajectories and appears to be partially mediated by systemic inflammation. Our findings suggest that improvements in insulin sensitivity are associated with a lower risk of depressive symptoms in older adults.

## Introduction

Depression is a common and debilitating mental disorder, affecting an estimated 350 million people worldwide. It is characterized by persistent low mood, anhedonia, and cognitive impairment (1, 2). The substantial disease burden of depression, including its association with an increased risk of cardiovascular disease and all-cause mortality, underscores the urgent need to identify modifiable risk factors (3). Emerging evidence suggests that complex interactions between metabolic dysregulation and mood disorders may play a key role in the pathophysiology of depression (4, 5).

Insulin resistance (IR), a core feature of type 2 diabetes mellitus (T2DM) and metabolic syndrome, has been implicated in the development and progression of depression (6, 7). A bidirectional relationship links these conditions: individuals with T2DM have approximately twice the risk of developing depression, while depression itself increases the incidence of T2DM by up to 60% (8–10). Meta-analytic evidence indicates that fasting insulin levels and the Homeostatic Model Assessment for Insulin Resistance (HOMA-IR) are elevated during acute depressive episodes and tend to normalize during remission, suggesting that IR may serve as a state-dependent biomarker. This pattern appears particularly pronounced in individuals with atypical depression, which is characterized by increased appetite and weight gain (11, 12).

The estimated glucose disposal rate (eGDR) is a practical, non-invasive surrogate arker of IR, calculated from waist circumference, hypertension status, and glycated hemoglobin (HbA1c) (13). Lower eGDR values reflect greater IR. Using data from the National Health and Nutrition Examination Survey (NHANES), Chen et al. (14) reported an inverse linear association between eGDR and depression, with individuals in the highest eGDR category (> 8 mg/kg/min) having 41.5% lower odds of depression than those in the lowest category (< 4 mg/kg/min). Similarly, in a prospective cohort analysis of the China Health and Retirement Longitudinal Study (CHARLS), distinct trajectories of eGDR were found to differentially predict depression risk. Compared with participants who had persistently low eGDR, those with a rapid decline in eGDR had a 23.2% higher risk of incident depression, whereas those with persistently high eGDR had a 16.2% lower risk of developing depression (15).

Most previous studies have focused on static eGDR measurements or predefined trajectory patterns, leaving a gap in understanding the dynamic interplay between concurrent changes in IR and depressive symptoms over time. It remains unclear whether the rates of change in eGDR are directly coupled with rates of change in depressive symptoms, and whether distinct patterns of co-evolution have different clinical implications. Addressing these questions requires analytical approaches that capture within-individual longitudinal associations, such as linear mixed models (LMM) with time-varying exposures.

Although the biological pathways linking IR to depression are not fully understood, chronic low-grade systemic inflammation has emerged as a key candidate mediator. IR promotes the production of pro-inflammatory cytokines, which in turn stimulate hepatic synthesis of C-reactive protein (CRP) (16). These inflammatory mediators can affect brain regions involved in mood regulation, including the hippocampus and prefrontal cortex (17, 18). Meta-analyses have confirmed elevated CRP levels in individuals with depression compared with controls (19, 20). In a large UK Biobank cohort, Jiang et al. (21) reported that inflammatory markers, particularly CRP, mediated the association between physical frailty and incident depression, a condition closely related to IR. However, no study to date has examined whether CRP mediates the longitudinal association between dynamic changes in eGDR and concurrent changes in depressive symptoms, or whether this mediating effect varies across different patterns of eGDR-depression co-evolution.

Against this background, the present study used data from the English Longitudinal Study of Ageing (ELSA) to (1): assess the longitudinal association between changes in eGDR and changes in depressive symptoms using LMM (2); explore potential nonlinear dose-response relationships between eGDR and depressive symptoms (3); test whether CRP mediates this association; and (4) examine whether these associations differ across subgroups defined by sociodemographic characteristics and clinical conditions. We hypothesized that declines in eGDR would be associated with increases in CES-D scores, with the strength of this association varying according to the direction of co-evolution. We further assumed that CRP would partially mediate this relationship. Clarifying these mechanisms could inform integrated metabolic-mental health strategies in aging populations.

## Materials and methods

### Study design and population

This study used data from ELSA, an ongoing multidisciplinary cohort of community-dwelling adults aged 50 years and older in England. Participants who provided blood samples at Wave 2 (2004–2005) were eligible for inclusion. Participants were sequentially excluded based on the following criteria (1): missing sociodemographic information (age, sex, marital status, or education level) at Wave 2 (2); loss to follow-up at Wave 4 (2008–2009) or Wave 6 (2012–2013) (3); missing data on the Center for Epidemiologic Studies Depression scale (CES-D), waist circumference, hypertension status, HbA1c, or CRP at any wave; or (4) CRP levels >10 mg/L, to minimize the influence of acute inflammatory conditions (22, 23). After applying these criteria, participants with complete data were included in the final analysis. All study variables were derived from the ELSA core dataset, including nurse-administered health assessments, self-completion questionnaires, and blood sample analyses conducted at Waves 2, 4, and 6.

### Assessment of exposure and outcome

IR was assessed using eGDR, calculated as:

eGDR (mg/kg/min) = 19.02 – [2.068 × waist circumference (inches)] – [0.806 × hypertension status (yes=1, no=0)] – [0.588 × HbA1c (%)].

Waist circumference was measured to the nearest 0.1 cm and converted to inches (1 inch=2.54 cm). Hypertension was defined as systolic blood pressure ≥140 mmHg, diastolic blood pressure ≥90 mmHg, or self-reported use of antihypertensive medication. HbA1c was measured from blood samples and expressed as a percentage. Lower eGDR values indicate more severe IR.

Depressive symptoms were assessed using CES-D scale. Participants reported whether they experienced specific feelings or behaviors during the past week, with each item scored 0 (no) or 1 (yes). Total scores ranged from 0 to 8, with higher scores indicating more severe depressive symptoms. CES-D score was analyzed as a continuous variable.

### Assessment of mediator and covariates

CRP was measured from fasting venous blood samples using high-sensitivity immunoturbidimetric assays. Due to skewed distribution, CRP values were natural log-transformed prior to analysis.

Covariates were selected based on prior literature and included:

Sociodemographic characteristics: age (continuous), gender (male/female), marital status (married/other), education level (below high school/high school/college or above);

Lifestyle factors: smoking status (never/former/current), drinking status (yes/no);

Chronic conditions: dyslipidemia, diabetes, heart disease, and stroke (all self-reported, physician-diagnosed);

Physical measurements: body mass index (BMI, kg/m²), systolic blood pressure (SBP, mmHg), and diastolic blood pressure (DBP, mmHg).

All covariates were derived from self-reported questionnaire data, except for BMI, SBP, and DBP, which were measured by trained nurses.

### Statistical analysis

Baseline characteristics of the study population were summarized using descriptive statistics. Continuous variables were presented as median with interquartile range (IQR), and categorical variables as counts with corresponding percentages. Participants were divided into quartiles based on eGDR levels. Between-group differences were assessed using the Kruskal–Wallis test for continuous variables and Pearson’s chi-square test for categorical variables.

To examine the longitudinal association between eGDR and CES-D scores, LMM were fitted. These models account for within-participant correlation of repeated measures and allow for time-varying exposures. Four sequential models were estimated: Model 1 was unadjusted; Model 2 adjusted for sociodemographic variables (age, sex, marital status, education level); Model 3 additionally adjusted for lifestyle factors (smoking, drinking); and Model 4 (fully adjusted) further adjusted for chronic conditions (dyslipidemia, diabetes, heart disease, stroke). Each model included time (years), eGDR (continuous), and an eGDR × time interaction term to test whether the rate of change in eGDR was associated with the rate of change in CES-D scores. Analyses were conducted in the total population and separately in each of the four trajectory subgroups.

To explore potential nonlinear dose-response relationships, we incorporated restricted cubic splines (RCS) with three knots at the 10th, 50th, and 90th percentiles into the LMM. We used the fully adjusted model (Model 4) as the base specification. Nonlinearity was assessed using a likelihood ratio test comparing models with and without the spline terms.

Mediation analysis was conducted to examine whether CRP mediated the association between eGDR and CES-D scores. The total effect was decomposed into an indirect effect (through CRP) and a direct effect (independent of CRP). The proportion mediated was calculated as (indirect effect/total effect) × 100%. Nonparametric bootstrap resampling with 1000 iterations was used to obtain 95% confidence intervals (CIs) for the indirect effect. Mediation was considered statistically significant if the CI did not include zero. All mediation analyses were performed in the overall study population and separately within each eGDR trajectory subgroup.

To assess whether the association between eGDR and CES-D scores varied across population subgroups, we performed subgroup analyses. Stratifying variables included age (50–60 years vs.≥60 years), sex, marital status, education level, smoking status, drinking status, dyslipidemia, diabetes, heart disease, and stroke. Within each stratum, LMM (Model 4) were fitted to estimate the association between eGDR and CES-D scores. Effect modification was assessed by adding a cross-product term between eGDR and each stratification variable to the model; a statistically significant interaction term (P < 0.05) was interpreted as evidence that the association between eGDR and CES-D scores differed across levels of the corresponding subgroup.

Sensitivity analyses were performed to assess the robustness of our findings. We conducted complete-case analyses by excluding participants with missing data at any wave. In addition, we applied multiple imputation by chained equations with an alternative random seed to handle missing data. All statistical analyses were performed using R software (version 4.2.1). Two-sided P values < 0.05 were considered statistically significant.

## Results

### Study population selection

**Figure 1:**
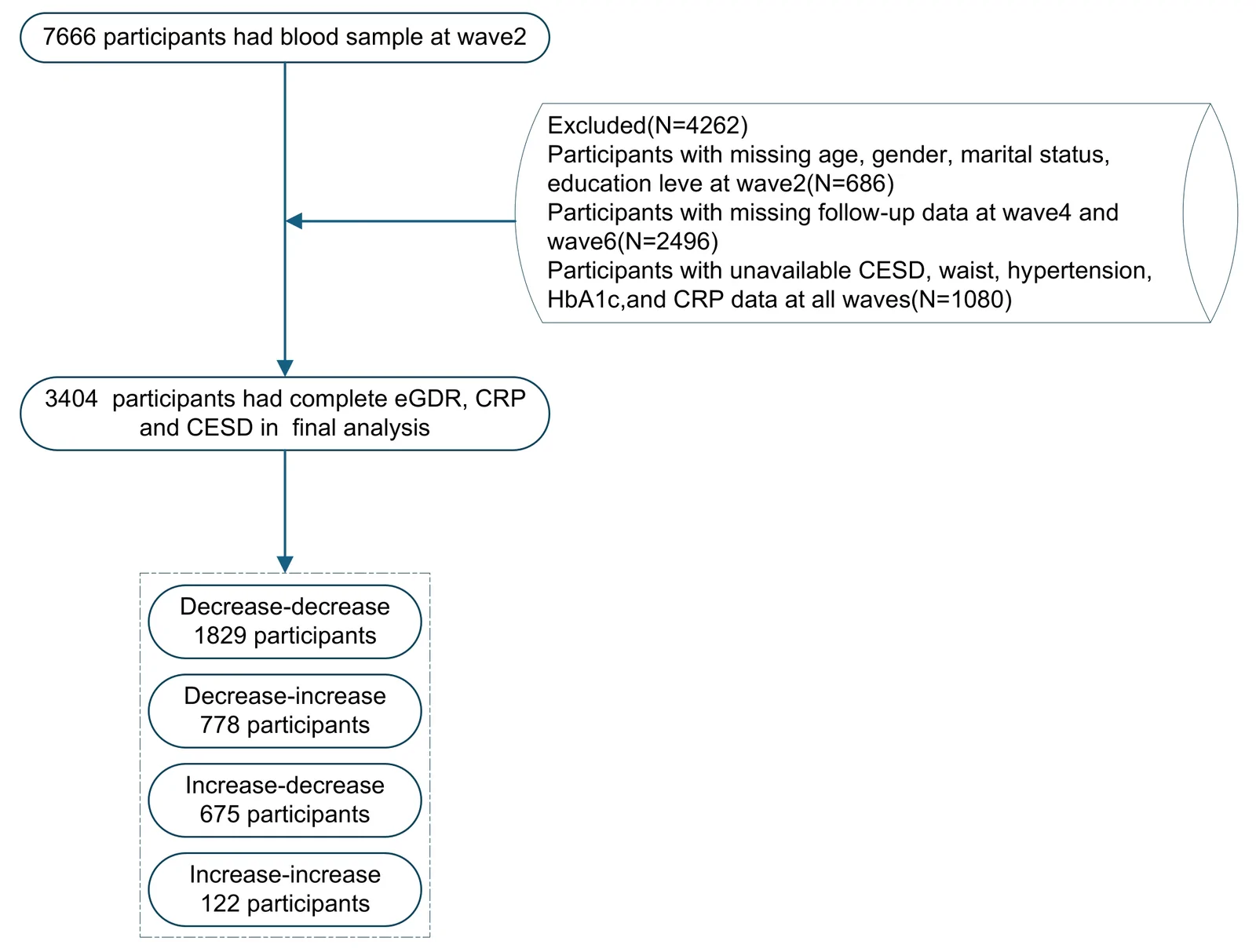
Flow chart of the study population.

The participant selection and grouping process is illustrated in **Figure 1**. At Wave 2 (2004–2005), 7,666 participants provided blood samples for CRP measurement. We sequentially excluded individuals with missing demographic covariates at Wave 2 (n = 686), those lost to follow-up at Wave 4 or Wave 6 (n = 2,496), and those with missing data on any core variable (CES-D score, waist circumference, hypertension status, HbA1c, or CRP) at any wave (n = 1,080). After these exclusions, 3,404 participants with complete data at all waves were included in the final analytic sample. The patterns and proportions of missing data are presented in **Supplementary Figure 1**.

Based on the direction of change in eGDR and CES-D scores from Wave 2 to Wave 6, participants were classified into four trajectory groups: decrease-decrease (eGDR decreased and CES-D decreased, n=1829), decrease-increase (eGDR decreased and CES-D increased, n=778), increase-decrease (eGDR increased and CES-D decreased, n=675), and increase-increase (eGDR increased and CES-D increased, n=122).

### Baseline characteristics

Baseline characteristics of the 3,404 participants are summarized in **Table 1**. Overall, 54% of participants were women, and the median age was 62 years (IQR: 57–69 years).

**Table 1:** Baseline characteristics by eGDR quartiles.

Characteristic	Overall	Quartile eDGR				P Value^2^
	Overall, N = 3404 (100%)^1^	Q1, N = 851 (25%)^1^	Q2, N = 851 (25%)^1^	Q3, N = 851 (25%)^1^	Q4, N = 851 (25%)^1^	
**Gender**						**<0.001**
*Male*	1,569 (46%)	475 (56%)	486 (57%)	320 (38%)	288 (34%)	
*Female*	1,835 (54%)	376 (44%)	365 (43%)	531 (62%)	563 (66%)	
**Age (years)**	62 (57, 69)	64 (58, 70)	63 (57, 70)	61 (57, 67)	62 (57, 70)	**<0.001**
**Marital status**						**<0.001**
*Others*	1,009 (30%)	259 (30%)	226 (27%)	227 (27%)	297 (35%)	
*Married*	2,395 (70%)	592 (70%)	625 (73%)	624 (73%)	554 (65%)	
**Education level**						**0.015**
*Below high school*	1,271 (37%)	356 (42%)	318 (37%)	281 (33%)	316 (37%)	
*College or above*	1,385 (41%)	333 (39%)	344 (40%)	363 (43%)	345 (41%)	
*High school*	748 (22%)	162 (19%)	189 (22%)	207 (24%)	190 (22%)	
**Smoking status**						**<0.001**
*Current Smoker*	442 (13%)	109 (13%)	98 (12%)	110 (13%)	125 (15%)	
*Former Smoker*	1,629 (48%)	427 (50%)	452 (53%)	365 (43%)	385 (45%)	
*Never Smoked*	1,333 (39%)	315 (37%)	301 (35%)	376 (44%)	341 (40%)	
**Drinking status**	2,952 (93%)	726 (92%)	730 (92%)	772 (95%)	724 (91%)	**0.004**
**BMI**	27.3 (24.7, 30.4)	29.4 (26.7, 32.3)	28.6 (26.2, 31.5)	25.7 (24.0, 27.5)	25.9 (22.7, 29.5)	**<0.001**
**SBP**	135 (123, 148)	141 (131, 154)	136 (125, 149)	130 (120, 143)	133 (120, 145)	**<0.001**
**DBP**	77 (70, 84)	80 (73, 87)	77 (71, 84)	74 (68, 82)	75 (67, 82)	**<0.001**
**Hypertension**	1,218 (36%)	810 (95%)	157 (18%)	19 (2.2%)	232 (27%)	**<0.001**
**Diabetes**	209 (6.1%)	107 (13%)	35 (4.1%)	16 (1.9%)	51 (6.0%)	**<0.001**
**Heart disease**	423 (12%)	138 (16%)	80 (9.4%)	71 (8.3%)	134 (16%)	**<0.001**
**Stroke**	92 (2.7%)	41 (4.8%)	17 (2.0%)	7 (0.8%)	27 (3.2%)	**<0.001**
**CRP**	1 (0, 3)	3 (1, 5)	2 (1, 4)	1 (1, 3)	-11 (-11, 0)	**<0.001**
**Dyslipidemia**	606 (18%)	206 (24%)	144 (17%)	109 (13%)	147 (17%)	**<0.001**
**FBG**	4.3 (-1.0, 4.9)	4.5 (-1.0, 5.1)	4.5 (-1.0, 5.1)	4.5 (-1.0, 4.9)	-1.0 (-1.0, -1.0)	**<0.001**
**TG**	1.3 (0.8, 2.0)	1.7 (1.2, 2.5)	1.7 (1.1, 2.3)	1.3 (0.9, 1.8)	-11.0 (-11.0, 0.8)	**<0.001**
**LDL-c**	3.3 (2.2, 4.1)	3.3 (2.5, 4.1)	3.6 (2.9, 4.3)	3.8 (3.2, 4.4)	-11.0 (-11.0, 3.0)	**<0.001**
**TC**	5.7 (4.4, 6.5)	5.7 (4.8, 6.5)	6.0 (5.2, 6.7)	6.1 (5.4, 6.8)	-11.0 (-11.0, 5.3)	**<0.001**
**HDL-c**	1.4 (1.1, 1.7)	1.4 (1.2, 1.6)	1.4 (1.2, 1.7)	1.6 (1.3, 1.8)	-11.0 (-11.0, 1.5)	**<0.001**
**HbA1c**	5.3 (5.0, 5.6)	5.6 (5.3, 5.9)	5.5 (5.2, 5.7)	5.3 (5.2, 5.6)	-11.0 (-11.0, 5.0)	**<0.001**
**Depression**	645 (19%)	179 (21%)	141 (17%)	136 (16%)	189 (22%)	**<0.001**

^1^ median (IQR) for continuous; n (%) for categorical.

^2^ Pearson's Chi-squared test; Kruskal-Wallis rank sum test. Bold values indicate statistical significance at P < 0.05.

When categorized by eGDR quartiles, all baseline characteristics differed significantly across groups (P < 0.001). Compared with participants in the highest eGDR quartile (Q4, indicating the greatest insulin sensitivity), those in the lowest eGDR quartile (Q1, indicating the most severe IR) displayed a more adverse metabolic profile, including higher BMI (29.4 vs. 25.9 kg/m²), systolic blood pressure (141 vs. 133 mmHg), triglyceride levels (1.7 vs. 0.8 mmol/L), and HbA1c levels (5.6% vs. 5.0%). The prevalences of hypertension (95% vs. 27%) and diabetes (13% vs. 6.0%) were also markedly higher in Q1 than in Q4.

Notably, the prevalence of heart disease showed a U-shaped distribution across eGDR quartiles (Q1: 16%, Q2: 9.4%, Q3: 8.3%, Q4: 16%). A similar U-shaped pattern was observed for depression defined by CES-D scores (Q1: 21%, Q2: 17%, Q3: 16%, Q4: 22%; P < 0.001), suggesting a potential nonlinear association between eGDR and these outcomes.

### Longitudinal association between eGDR and CES-D scores

LMM were fitted to examine the longitudinal association between eGDR and CES-D scores, with results presented in **Table 2**. Models were sequentially adjusted for sociodemographic characteristics (Model 2), lifestyle factors (Model 3), and chronic conditions (Model 4).

**Table 2:** Linear mixed models for the association between the cumulative eGDR index and CES-D.

Categories	Model 1			Model 2			Model 3			Model 4		P-value
	Beta	95% CI^1^	P-value	Beta	95% CI	P-value	Beta	95% CI	P-value	Beta	95% CI	
All trend												
Time, years	-0.022	-0.048,0.004	0.101	-0.039	-0.067, -0.011	**0.007**	-0.106	-0.143, -0.068	**<0.001**	-0.102	-0.140, -0.064	**<0.001**
Continuous eGDR	0.030	0.011,0.049	**0.002**	0.023	0.004, 0.042	**0.019**	-0.030	-0.060, 0.000	0.054	-0.022	-0.052, 0.008	0.156
Continuous eGDR × Time	-0.005	-0.009, -0.002	**0.001**	-0.004	-0.008, -0.001	**0.009**	0.003	-0.001, 0.008	0.172	0.002	-0.003, 0.007	0.349
Decrease-decrease												
Time, years	-0.262	-0.288, -0.236	**<0.001**	-0.270	-0.299, -0.242	**<0.001**	-0.309	-0.347, -0.271	**<0.001**	-0.310	-0.349, -0.272	**<0.001**
Continuous eGDR	0.016	-0.003, 0.035	0.104	0.016	-0.003, 0.035	0.101	0.0003	-0.032, 0.031	0.985	-0.001	-0.033, 0.030	0.939
Continuous eGDR × Time	-0.004	-0.007, -0.001	**0.018**	-0.004	-0.007, -0.001	**0.016**	-0.002	-0.007, 0.003	0.442	-0.002	-0.007, 0.003	0.469
Decrease-increase												
Time, years	0.082	0.026, 0.138	**0.004**	0.082	0.021, 0.142	**0.008**	-0.047	-0.124, 0.030	0.233	-0.050	-0.128, 0.028	0.208
Continuous eGDR	0.061	0.021, 0.101	**0.003**	0.059	0.019, 0.098	**0.004**	0.038	-0.023, 0.099	0.219	0.032	-0.029, 0.093	0.310
Continuous eGDR × Time	-0.020	-0.027, -0.013	**<0.001**	-0.020	-0.026, -0.013	**<0.001**	-0.019	-0.029, -0.009	**<0.001**	-0.018	-0.028, -0.008	**<0.001**
Increase-decrease												
Time, years	0.287	0.228, 0.345	**<0.001**	0.286	0.222, 0.350	**<0.001**	0.332	0.248, 0.416	**<0.001**	0.337	0.253, 0.421	**<0.001**
Continuous eGDR	0.004	-0.038, 0.046	0.850	-0.004	-0.046, 0.038	0.864	-0.072	-0.137, -0.007	**0.030**	-0.039	-0.104, 0.026	0.234
Continuous eGDR × Time	0.011	0.004, 0.019	**0.002**	0.012	0.005, 0.020	**<0.001**	0.025	0.015, 0.036	**<0.001**	0.021	0.010, 0.031	**<0.001**
Increase-increase												
Time, years	0.287	0.228, 0.345	**<0.001**	0.879	0.785, 0.972	**<0.001**	0.895	0.778, 1.01	**<0.001**	0.890	0.771, 1.01	**<0.001**
Continuous eGDR	0.004	-0.038, 0.046	0.850	-0.003	-0.053, 0.047	0.900	0.023	-0.061, 0.107	0.595	0.022	-0.063, 0.108	0.604
Continuous eGDR × Time	0.011	0.004, 0.019	**0.002**	-0.001	-0.010, 0.007	0.751	-0.006	-0.020, 0.008	0.385	-0.006	-0.020, 0.008	0.391

CI, Confidence Interval, β-coefficient (95% CI). Bold values indicate statistical significance at P < 0.05.

In the total population, the fully adjusted model (Model 4) showed a significant negative association between time and CES-D score (β = -0.102, 95% CI: -0.140 to -0.064, P < 0.001), indicating an overall decline in depressive symptoms over follow-up. Neither the main effect of eGDR (β = -0.022, 95% CI: -0.052 to 0.008, P = 0.156) nor the interaction between eGDR and time (β = 0.002, 95% CI: -0.003 to 0.007, P = 0.349) was statistically significant.

Stratified analyses by eGDR trajectory pattern revealed marked heterogeneity in the eGDR × time interaction. In the decrease-increase group, the interaction was significantly negative (β = -0.018, 95% CI: -0.028 to -0.008, P < 0.001), suggesting that a faster decline in eGDR was associated with a faster increase in CES-D scores. In the increase-decrease group, the interaction was significantly positive (β = 0.021, 95% CI: 0.010 to 0.031, P < 0.001), indicating that a faster increase in eGDR was associated with a faster decline in CES-D scores. In the decrease-decrease (β = -0.002, 95% CI: -0.007 to 0.003, P = 0.469) and increase-increase groups (β = -0.006, 95% CI: -0.020 to 0.008, P = 0.391), the eGDR × time interaction was not statistically significant.

The main effect of time was significant in the decrease-decrease (β = -0.310, P < 0.001), increase-decrease (β = 0.337, P < 0.001), and increase-increase groups (β = 0.890, P < 0.001), but not in the decrease-increase group (β = -0.050, P = 0.208). The main effect of eGDR was not statistically significant in any subgroup (all P > 0.05).

### Dose-response relationship between eGDR and CES-D scores

**Figure 2:**
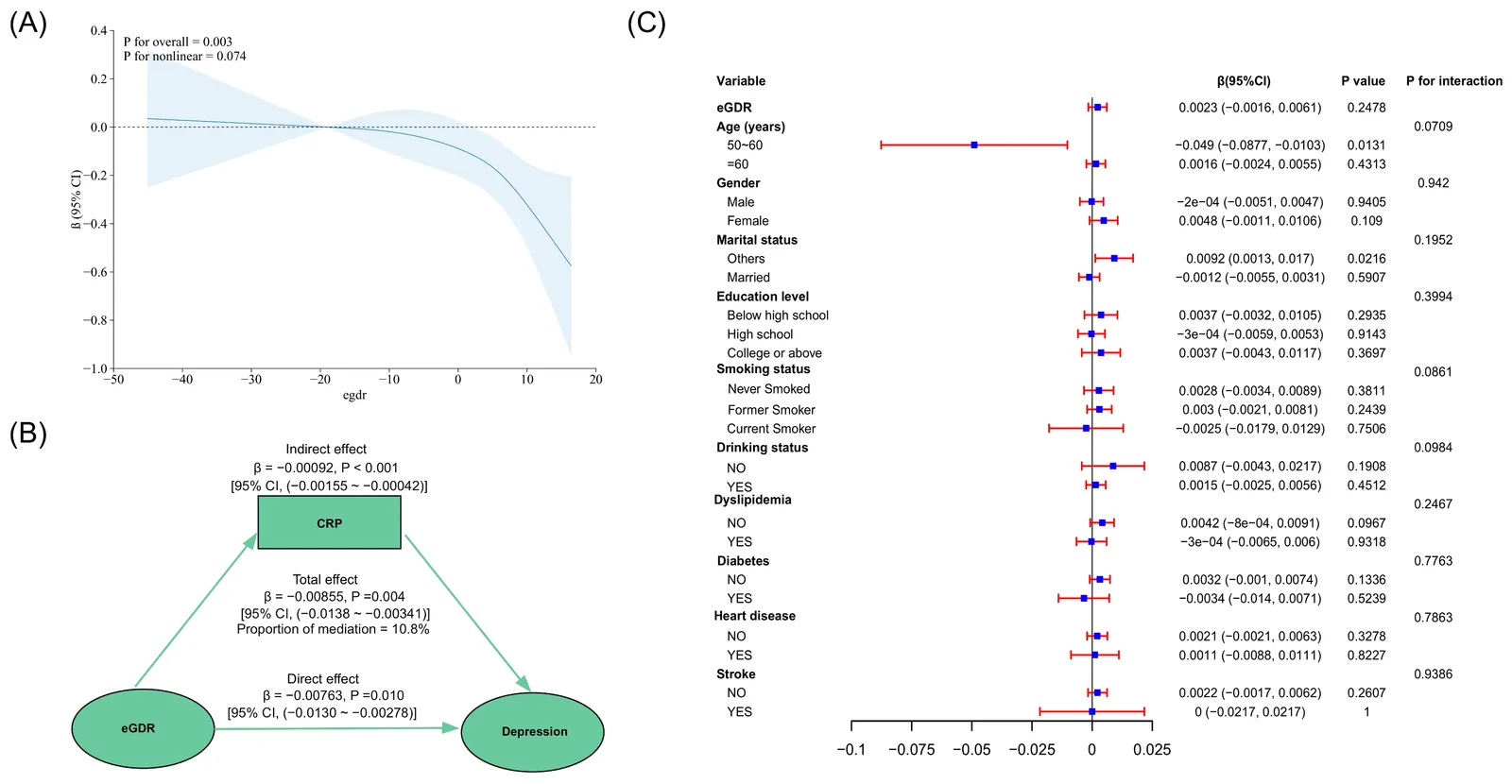
Association between eGDR and CES-D scores. **(A)** RCS plot showing the dose-response relationship. **(B)** Mediation analysis of CRP. **(C)** Results of subgroup analyses.

To further explore the dose–response relationship, RCS were incorporated into the linear mixed model (**Figure 2A**). A significant overall association was observed between eGDR and CES-D scores (P = 0.003), with lower eGDR values associated with progressively higher CES-D scores. The test for nonlinearity indicated no statistically significant departure from linearity (P = 0.074).

As shown in **Supplementary Figure 2**, subgroup dose-response analyses revealed marked heterogeneity across trajectory groups. In the decrease-decrease group, the overall association was significant (P < 0.001), and the test for nonlinearity was also significant (P = 0.013). In the decrease-increase group, both the overall association (P < 0.001) and nonlinearity (P < 0.001) were significant. In the increase-decrease group, the overall association (P < 0.001) and nonlinearity (P = 0.002) were also significant. In contrast, in the increase-increase group, neither the overall association (P = 0.383) nor nonlinearity (P = 0.234) was significant.

### Mediation, subgroup, and sensitivity analyses

#### Mediating role of CRP

The mediating effect of CRP on the association between eGDR and CES-D was evaluated using causal mediation analysis based on bootstrap resampling. As shown in **Figure 2B**, after adjustment for all covariates, the total effect of eGDR on CES-D was -0.00855 (95% CI: -0.0138 to -0.00341, P = 0.004). The indirect effect through CRP was -0.00092 (95% CI: -0.00155 to -0.00042, P < 0.001), while the direct effect of eGDR on CES-D, accounting for CRP, was -0.00763 (95% CI: -0.0130 to -0.00278, P = 0.010). CRP mediated 10.8% of the total effect of eGDR on CES-D.

### Subgroup and sensitivity analyses

Results of subgroup analyses are presented in **Figure 2C**. All interaction P values were >0.05, indicating no significant heterogeneity across the stratified variables.

In participants aged 50–60 years, eGDR was significantly and inversely associated with CES-D scores (β = -0.049, 95% CI: -0.088 to -0.010, P = 0.013), whereas no significant association was observed in those aged ≥60 years (β = 0.002, 95% CI: -0.002 to 0.006, P = 0.431). However, the age × eGDR interaction was not statistically significant (P = 0.071). Among participants with “other” marital status (unmarried, divorced, or widowed), eGDR was positively associated with CES-D (β = 0.009, 95% CI: 0.001 to 0.017, P = 0.022), but not among married participants (β = -0.001, 95% CI: -0.006 to 0.003, P = 0.591). The marital status × eGDR interaction was not significant (P = 0.195). In all other subgroups, the associations between eGDR and CES-D were not significant (all P > 0.05), and none of the interaction terms reached statistical significance (all P > 0.05).

To further investigate potential effect modification, all covariates and their interaction terms with eGDR were included simultaneously in a single LMM. Results are presented in **Supplementary Table 1**. In this model, significant interactions were observed for eGDR × alcohol consumption (β = -0.009, 95% CI: -0.017 to -0.001, P = 0.037), eGDR × age (β = -0.0003, 95% CI: -0.0005 to -0.0001, P = 0.010), and eGDR × marital status (β = -0.006, 95% CI: -0.012 to 0.000, P = 0.035). No other interaction terms were statistically significant (all P > 0.05).

Sensitivity analyses confirmed the robustness of the findings. In each trajectory subgroup, the direction and statistical significance of the eGDR × time interaction were consistent across complete-case analyses (**Supplementary Table 2**) and multiple imputation analyses with a different random seed (**Supplementary Table 3**).

## Discussion

In this longitudinal study of 3404 community-dwelling older adults from ELSA, we investigated the association between IR, measured by eGDR, and depressive symptoms, assessed using the CES-D, over an 8-year follow-up. We also examined systemic inflammation measured by CRP, as a potential mediator of this association and explored heterogeneity across distinct eGDR trajectory subgroups.

In the total population, eGDR was not significantly associated with CES-D scores over time. However, significant associations emerged when participants were stratified by joint trajectories of eGDR and CES-D. In the decrease-increase group, a faster decline in eGDR was associated with a faster rise in depressive symptoms. Conversely, in the increase-decrease group, a faster improvement in insulin sensitivity was linked to a faster decline in depressive symptoms. These findings suggest that dynamic changes in IR, rather than static baseline levels, are more closely related to the evolution of depressive symptoms.

RCS analyses revealed a significant overall inverse association between eGDR and CES-D scores, with no evidence of nonlinearity. However, subgroup analyses demonstrated nonlinear associations in several trajectory groups, particularly the decrease-increase and increase-decrease groups. These findings suggest that the relationship between IR and depressive symptoms may vary across trajectory subgroups, reflecting heterogeneity in the dynamic interplay between metabolic and mental health.

Mediation analysis showed that CRP accounted for approximately 10.8% of the association between eGDR and CES-D scores, supporting the hypothesis that systemic inflammation partially links insulin resistance and depressive symptoms. IR is known to promote a pro-inflammatory state through adipokine dysregulation and oxidative stress, which in turn may affect neurotransmitter metabolism and neuroendocrine function (16, 24, 25). However, the relatively modest mediation effect indicates that CRP represents only one component of a much more complex biological pathway. Other mechanisms, including hypothalamic-pituitary-adrenal (HPA) axis dysregulation, endothelial dysfunction, gut microbiome alterations, impaired insulin signaling in brain regions involved in mood regulation, and psychosocial factors such as social isolation and socioeconomic stress, are also likely to contribute (26). Future studies integrating multi-omics approaches and comprehensive psychosocial assessments are needed to clarify the relative contributions of these interconnected pathways.

Subgroup analyses revealed no statistically significant interactions across most stratifying variables, suggesting that the association between eGDR and CES-D is generally consistent across demographic and clinical groups. However, in the fully adjusted model that included interaction terms, we observed significant effect modification by age, alcohol consumption, and marital status. These findings should be interpreted with caution given the exploratory nature of these analyses and the lack of correction for multiple testing. Nevertheless, they point to potential subgroups, such as individuals aged 50–60 years, non-drinkers, and unmarried participants, who may be more vulnerable to the mental health impacts of IR.

Our findings are consistent with previous cross-sectional and longitudinal studies reporting an association between IR and depressive symptoms (27). However, most previous studies have relied on HOMA-IR or fasting insulin to estimate IR (28). eGDR integrates waist circumference, hypertension, and HbA1c, providing a practical surrogate for large epidemiological studies (13). Compared with HOMA-IR, eGDR does not require insulin assays and incorporates multiple clinical components related to metabolic dysfunction. Compared with the TyG index, which is calculated solely from fasting triglycerides and glucose, eGDR additionally incorporates information on central obesity and hypertension. In the present study, eGDR was selected because fasting insulin measurements were unavailable in the ELSA cohort and because its multidimensional nature made it well suited for evaluating longitudinal changes in metabolic health. The trajectory-based approach we employed extends prior work by showing that the dynamic interplay between metabolic and mental health over time matters more than static measures.

This study has several strengths, including its longitudinal design, large sample size, use of validated measures, and rigorous statistical approaches, including LMM, RCS, and causal mediation analysis. The stratification by joint trajectories provided novel insights into the heterogeneity of the relationship between eGDR and CES-D.

Several limitations should be acknowledged. First, despite adjusting for a wide range of covariates, residual confounding cannot be ruled out. Specifically, we lacked data on several potentially important confounders, including antidepressant medication use, objective measures of physical activity, detailed dietary habits, and socioeconomic indicators beyond educational level (e.g., income, occupation, wealth). These factors are independently associated with both metabolic health and depressive symptoms, and may have influenced our effect estimates. Additionally, we did not account for other chronic inflammatory conditions, which could elevate CRP levels independently of IR and potentially confound the observed mediation pathway. Although diabetes status was included as a covariate and HbA1c was incorporated into the eGDR calculation, information on specific glucose-lowering therapies and medications that may influence CRP levels, such as statins and anti-inflammatory agents, was not included in the primary models. These unmeasured factors could have contributed to residual confounding. Second, depressive symptoms were assessed using the CES-D scale, a well-validated screening tool for epidemiological research, rather than a structured clinical diagnostic interview such as the Structured Clinical Interview for DSM Disorders (SCID). While the CES-D performs well in identifying probable depression in community-based populations, it does not substitute for a clinical diagnosis of major depressive disorder. Some degree of outcome misclassification is possible, as participants with elevated CES-D scores may not meet diagnostic criteria for clinical depression, whereas some individuals with clinically significant depression may have scored below the threshold. Such non-differential misclassification would likely attenuate the observed associations towards the null, suggesting that our effect estimates may be conservative. Third, CRP was measured only at baseline, limiting our ability to assess time-varying mediation. Fourth, the exclusion of participants with missing data may introduce selection bias, although sensitivity analyses using multiple imputation yielded similar results. Fifth, the classification of participants into four trajectory groups based solely on the direction of change in eGDR and CES-D scores represents a methodological simplification. Although more sophisticated approaches such as latent class trajectory modelling or group-based trajectory modelling are available, this approach was adopted because of its clinical interpretability, transparency, and consistency with our primary objective of examining dynamic associations using linear mixed models with time-varying exposures. However, it does not account for the magnitude, rate, or shape of change over time, nor does it capture potentially distinct trajectories within the same direction category. Future studies employing more advanced trajectory modelling methods are needed to validate and extend our findings. Finally, the observational nature of the study precludes causal inference, and the observed associations should therefore be interpreted with appropriate caution.

## Conclusions

This longitudinal study demonstrates that the relationship between IR and depressive symptoms in older adults is dynamic and trajectory-dependent. Changes in eGDR are associated with corresponding changes in CES-D scores in specific subgroups, and this association is partially mediated by systemic inflammation. Future research should clarify the underlying biological mechanisms and examine whether changes in insulin sensitivity are prospectively associated with the burden of depression in at-risk individuals.

## Data Availability

The original contributions presented in the study are included in the article/**Supplementary Material**. Further inquiries can be directed to the corresponding author.
