# Supplementary material for: Longitudinal association between insulin resistance and depressive symptoms in older adults: the mediating role of inflammation in the ELSA cohort
Source: Front Psychiatry. 2026 Aug 12;17:1848018. doi: 10.3389/fpsyt.2026.1848018 (PMC13506832; doi:10.3389/fpsyt.2026.1848018)
Supplement: Supplementary file 1 [file Table1.docx]

Supplementary Material

# 1 Supplementary Figures and Tables

## 1.1 Supplementary Figures

**Supplementary Figure 1.** Patterns and proportions of missing data.

**Supplementary Figure 2.** Subgroup dose-response analyses of the association between eGDR and CES-D scores stratified by trajectory groups. (A) Decrease-decrease group. (B) Decrease-increase group. (C) Increase-decrease group. (D) Increase-increase group.

1.2 **Supplementary Tables**

**Table S1. Longitudinal association of variable with CESD-10 (linear mixed model).**

| **Variable** | **Beta** | **95% CI** | **p-value** |
| --- | --- | --- | --- |
| **egdr** | 0.039 | -0.009, 0.088 | 0.111 |
| **time** | -0.110 | -0.149, -0.072 | **<0.001** |
| **agey** | -0.004 | -0.010, 0.002 | 0.246 |
| **mstat** | -0.516 | -0.608, -0.424 | **<0.001** |
| **education** | -0.133 | -0.195, -0.072 | **<0.001** |
| **ragender** | 0.473 | 0.378, 0.568 | **<0.001** |
| **smoke** | 0.185 | 0.118, 0.252 | **<0.001** |
| **drink** | -0.357 | -0.480, -0.234 | **<0.001** |
| **newhdl** | -0.027 | -0.085, 0.030 | 0.351 |
| **newcho** | -0.037 | -0.075, 0.002 | 0.064 |
| **newcrp** | 0.006 | 0.001, 0.012 | **0.021** |
| **newldl** | 0.009 | -0.023, 0.040 | 0.595 |
| **newtg** | 0.034 | -0.007, 0.074 | 0.104 |
| **newglu** | 0.002 | -0.007, 0.011 | 0.663 |
| **hchole** | 0.086 | 0.005, 0.167 | **0.038** |
| **diabe** | 0.204 | 0.058, 0.351 | **0.006** |
| **hearte** | 0.250 | 0.143, 0.357 | **<0.001** |
| **stroke** | 0.396 | 0.199, 0.593 | **<0.001** |
| **egdr * time** | 0.004 | -0.002, 0.009 | 0.163 |
| **egdr * agey** | 0.000 | -0.001, 0.000 | **0.010** |
| **egdr * mstat** | -0.006 | -0.012, 0.000 | **0.035** |
| **egdr * education** | -0.001 | -0.005, 0.003 | 0.644 |
| **egdr * ragender** | 0.000 | -0.006, 0.005 | 0.874 |
| **egdr * smoke** | -0.004 | -0.008, 0.000 | 0.076 |
| **egdr * drink** | -0.009 | -0.017, -0.001 | **0.037** |
| **egdr * newhdl** | 0.003 | -0.001, 0.007 | 0.172 |
| **egdr * newcho** | 0.000 | -0.004, 0.004 | 0.915 |
| **egdr * newcrp** | 0.000 | -0.001, 0.000 | 0.115 |
| **egdr * newldl** | -0.003 | -0.007, 0.000 | 0.072 |
| **egdr * newtg** | 0.000 | -0.003, 0.004 | 0.912 |
| **egdr * newglu** | 0.000 | -0.001, 0.001 | 0.898 |
| **egdr * hchole** | -0.003 | -0.009, 0.003 | 0.281 |
| **egdr * diabe** | 0.000 | -0.007, 0.008 | 0.932 |
| **egdr * hearte** | 0.003 | -0.004, 0.010 | 0.437 |
| **egdr * stroke** | -0.002 | -0.015, 0.010 | 0.701 |
| Abbreviations: CI = Confidence Interval, β-coefficient (95% CI) | | | |

**Table S2. Linear mixed models for the association between the cumulative eGDR index and CES-D (complete-case analysis).**

| **Categories** | | | | | | **Model 1** | | | **Model 2** | | | | | | | | | **Model 3** | | | | | | | | | **Model 4** | | | | | | |
| --- | --- | --- | --- | --- | --- | --- | --- | --- | --- | --- | --- | --- | --- | --- | --- | --- | --- | --- | --- | --- | --- | --- | --- | --- | --- | --- | --- | --- | --- | --- | --- | --- | --- |
|  | | | **Beta** | | **95% CI^1^** | | **p-value** | **Beta** | | | **95% CI** | | **p-value** | | | **Beta** | | | **95% CI** | | **p-value** | | | | **Beta** | | | **95% CI** | | **p-value** | | |  |
| **All trend** | |  | |  | | |  |  | |  | | | |  | |  | | |  | | |  | |  | | | |  | | |  | |  |
| **Time, years** | | -0.028 | | -0.059, 0.002 | | | 0.067 | -0.042 | | -0.075, -0.009 | | | | **0.012** | | -0.110 | | | -0.153, -0.066 | | | **<0.001** | | -0.107 | | | | -0.151, -0.063 | | | **<0.001** | |  |
| **Continuous eGDR** | | 0.033 | | 0.011, 0.056 | | | **0.004** | .024 | | 0.002, 0.047 | | | | **0.033** | | -0.036 | | | -0.071, -0.001 | | | **0.044** | | -0.028 | | | | -0.063, 0.007 | | | 0.114 | |  |
| **Continuous eGDR** × **Time** | | -0.006 | | -0.010, -0.002 | | | **0.001** | -0.005 | | -0.009, -0.001 | | | | **0.013** | | 0.004 | | | -0.001, 0.010 | | | 0.146 | | 0.003 | | | | -0.003, 0.009 | | | 0.278 | |  |
| **Decrease-decrease** | |  | |  | | |  |  | |  | | | |  | |  | | |  | | |  | |  | | | |  | | |  | |  |
| **Time, years** | | -0.247 | | -0.277, -0.218 | | | **<0.001** | -0.252 | | -0.284, -0.220 | | | | **<0.001** | | -0.300 | | | -0.344, -0.257 | | | **<0.001** | | -0.303 | | | | -0.347, -0.260 | | | **<0.001** | |  |
| **Continuous eGDR** | | 0.016 | | -0.005, 0.038 | | | 0.140 | 0.017 | | -0.005, 0.038 | | | | 0.136 | | -0.009 | | | -0.045, 0.027 | | | 0.621 | | -0.010 | | | | -0.046, 0.026 | | | 0.594 | |  |
| **Continuous eGDR** × **Time** | | -0.004 | | -0.008, 0.000 | | | **0.035** | -0.004 | | -0.008, 0.000 | | | | **0.032** | | -0.001 | | | -0.006, 0.005 | | | 0.835 | | -0.001 | | | | -0.006, 0.005 | | | 0.848 | |  |
| **Decrease-increase** | |  | |  | | |  |  | |  | | | |  | |  | | |  | | |  | |  | | | |  | | |  | |  |
| **Time, years** | | 0.040 | | -0.029, 0.109 | | | 0.252 | 0.060 | | -0.014, 0.134 | | | | 0.111 | | -0.039 | | | -0.131, 0.054 | | | 0.414 | | -0.046 | | | | -0.139, 0.048 | | | 0.341 | |  |
| **Continuous eGDR** | | 0.059 | | 0.011, 0.108 | | | **0.017** | 0.051 | | 0.003, 0.099 | | | | **0.038** | | 0.029 | | | -0.043, 0.101 | | | 0.428 | | 0.022 | | | | -0.050, 0.095 | | | 0.551 | |  |
| **Continuous eGDR** × **Time** | | -0.021 | | -0.029, -0.013 | | | **<0.001** | -0.020 | | -0.028, -0.011 | | | | **<0.001** | | -0.018 | | | -0.030, -0.006 | | | **0.003** | | -0.017 | | | | -0.029, -0.005 | | | **0.004** | |  |
| **Increase-decrease** | |  | |  | | |  |  | |  | | | |  | |  | | |  | | |  | |  | | | |  | | |  | |  |
| **Time, years** | | 0.319 | | 0.249, 0.390 | | | **<0.001** | 0.312 | | 0.234, 0.389 | | | | **<0.001** | | 0.353 | | | 0.252, 0.454 | | | **<0.001** | | 0.357 | | | | 0.257, 0.458 | | | **<0.001** | |  |
| **Continuous eGDR** | | 0.025 | | -0.027, 0.077 | | | 0.341 | 0.020 | | -0.032, 0.072 | | | | 0.443 | | -0.055 | | | -0.133, 0.024 | | | 0.175 | | -0.020 | | | | -0.099, 0.059 | | | 0.627 | |  |
| **Continuous eGDR** × **Time** | | 0.009 | | 0.000, 0.018 | | | 0.060 | 0.009 | | 0.000, 0.018 | | | | **0.043** | | 0.023 | | | 0.011, 0.036 | | | **<0.001** | | 0.018 | | | | 0.006, 0.031 | | | **0.0048** | |  |
| **Increase-increase** | |  | |  | | |  |  | |  | | | |  | |  | | |  | | |  | |  | | | |  | | |  | |  |
| **Time, years** | | 0.831 | | 0.729, 0.934 | | | **<0.001** | 0.854 | | 0.744, 0.965 | | | | **<0.001** | | 0.876 | | | 0.744, 1.01 | | | **<0.001** | | 0.861 | | | | 0.728, 0.995 | | | **<0.001** | |  |
| **Continuous eGDR** | | -0.027 | | -0.082, 0.027 | | | 0.318 | -0.041 | | -0.095, 0.012 | | | | 0.129 | | -0.009 | | | -0.099, 0.080 | | | 0.836 | | -0.004 | | | | -0.094, 0.086 | | | 0.930 | |  |
| **Continuous eGDR** × **Time** | | 0.002 | | -0.007, 0.012 | | | 0.660 | 0.004 | | -0.005, 0.014 | | | | 0.347 | | -0.001 | | | -0.016, 0.014 | | | 0.886 | | -0.002 | | | | -0.017, 0.013 | | | 0.782 | |  |
|  | CI = Confidence Interval, β-coefficient (95% CI) | | | | | | | |  | | |  | | |  | |  | | |  | | |  | | |  | | |  | | |  | |

**Table S3. Linear mixed models for the association between the cumulative eGDR index and CES-D (multiple imputation analysis).**

| **Categories** | | | | | | **Model 1** | | | **Model 2** | | | | | | | | | **Model 3** | | | | | | | | | **Model 4** | | | | | | |
| --- | --- | --- | --- | --- | --- | --- | --- | --- | --- | --- | --- | --- | --- | --- | --- | --- | --- | --- | --- | --- | --- | --- | --- | --- | --- | --- | --- | --- | --- | --- | --- | --- | --- |
|  | | | **Beta** | | **95% CI^1^** | | **p-value** | **Beta** | | | **95% CI** | | **p-value** | | | **Beta** | | | **95% CI** | | **p-value** | | | | **Beta** | | | **95% CI** | | **p-value** | | |  |
| **All trend** | |  | |  | | |  |  | |  | | | |  | |  | | |  | | |  | |  | | | |  | | |  | |  |
| **Time, years** | | -0.022 | | -0.048,0.004 | | | 0.101 | -0.039 | | -0.067, -0.011 | | | | **0.007** | | -0.106 | | | -0.143, -0.069 | | | **<0.001** | | -0.102 | | | | -0.140, -0.065 | | | **<0.001** | |  |
| **Continuous eGDR** | | 0.030 | | 0.011,0.049 | | | **0.002** | 0.023 | | 0.004, 0.042 | | | | **0.019** | | -0.030 | | | -0.060, 0.000 | | | 0.053 | | -0.022 | | | | -0.052, 0.008 | | | 0.155 | |  |
| **Continuous eGDR** × **Time** | | -0.005 | | -0.009, -0.002 | | | **0.001** | -0.004 | | -0.008, -0.001 | | | | **0.009** | | 0.003 | | | -0.001, 0.008 | | | 0.170 | | 0.002 | | | | -0.003, 0.007 | | | 0.347 | |  |
| **Decrease-decrease** | |  | |  | | |  |  | |  | | | |  | |  | | |  | | |  | |  | | | |  | | |  | |  |
| **Time, years** | | -0.262 | | -0.288, -0.236 | | | **<0.001** | -0.270 | | -0.299, -0.242 | | | | **<0.001** | | -0.309 | | | -0.347, -0.271 | | | **<0.001** | | -0.310 | | | | -0.349, -0.272 | | | **<0.001** | |  |
| **Continuous eGDR** | | 0.016 | | -0.003, 0.035 | | | 0.104 | 0.016 | | -0.003, 0.035 | | | | 0.101 | | 0.0003 | | | -0.032, 0.031 | | | 0.981 | | -0.001 | | | | -0.033, 0.030 | | | 0.935 | |  |
| **Continuous eGDR** × **Time** | | -0.004 | | -0.007, -0.001 | | | **0.018** | -0.004 | | -0.007, -0.001 | | | | **0.016** | | -0.002 | | | -0.007, 0.003 | | | 0.445 | | -0.002 | | | | -0.007, 0.003 | | | 0.472 | |  |
| **Decrease-increase** | |  | |  | | |  |  | |  | | | |  | |  | | |  | | |  | |  | | | |  | | |  | |  |
| **Time, years** | | 0.082 | | 0.026, 0.138 | | | **0.004** | 0.082 | | 0.021, 0.142 | | | | **0.008** | | -0.047 | | | -0.124, 0.030 | | | 0.233 | | -0.050 | | | | -0.128, 0.028 | | | 0.210 | |  |
| **Continuous eGDR** | | 0.061 | | 0.021, 0.101 | | | **0.003** | 0.059 | | 0.019, 0.098 | | | | **0.004** | | 0.038 | | | -0.023, 0.098 | | | 0.224 | | 0.031 | | | | -0.030, 0.093 | | | 0.314 | |  |
| **Continuous eGDR** × **Time** | | -0.020 | | -0.027, -0.013 | | | **<0.001** | -0.020 | | -0.026, -0.013 | | | | **<0.001** | | -0.019 | | | -0.029, -0.009 | | | **<0.001** | | -0.018 | | | | -0.028, -0.008 | | | **<0.001** | |  |
| **Increase-decrease** | |  | |  | | |  |  | |  | | | |  | |  | | |  | | |  | |  | | | |  | | |  | |  |
| **Time, years** | | 0.287 | | 0.228, 0.345 | | | **<0.001** | 0.286 | | 0.222, 0.350 | | | | **<0.001** | | 0.332 | | | 0.248, 0.416 | | | **<0.001** | | 0.337 | | | | 0.253, 0.421 | | | **<0.001** | |  |
| **Continuous eGDR** | | 0.004 | | -0.038, 0.046 | | | 0.850 | -0.004 | | -0.046, 0.038 | | | | 0.864 | | -0.072 | | | -0.137, -0.007 | | | **0.030** | | -0.039 | | | | -0.104, 0.026 | | | 0.235 | |  |
| **Continuous eGDR** × **Time** | | 0.011 | | 0.004, 0.019 | | | **0.002** | 0.012 | | 0.005, 0.020 | | | | **<0.001** | | 0.025 | | | 0.015, 0.036 | | | **<0.001** | | 0.021 | | | | 0.010, 0.031 | | | **<0.001** | |  |
| **Increase-increase** | |  | |  | | |  |  | |  | | | |  | |  | | |  | | |  | |  | | | |  | | |  | |  |
| **Time, years** | | 0.852 | | 0.770, 0.935 | | | **<0.001** | 0.879 | | 0.785, 0.972 | | | | **<0.001** | | 0.895 | | | 0.778, 1.01 | | | **<0.001** | | 0.890 | | | | 0.771, 1.01 | | | **<0.001** | |  |
| **Continuous eGDR** | | 0.005 | | -0.045, 0.056 | | | 0.831 | -0.003 | | -0.053, 0.047 | | | | 0.900 | | 0.023 | | | -0.061, 0.107 | | | 0.595 | | 0.022 | | | | -0.063, 0.108 | | | 0.604 | |  |
| **Continuous eGDR** × **Time** | | -0.003 | | -0.012, 0.006 | | | 0.533 | -0.001 | | -0.010, 0.007 | | | | 0.751 | | -0.006 | | | -0.020, 0.008 | | | 0.385 | | -0.006 | | | | -0.020, 0.008 | | | 0.391 | |  |
|  | CI = Confidence Interval, β-coefficient (95% CI) | | | | | | | |  | | |  | | |  | |  | | |  | | |  | | |  | | |  | | |  | |
